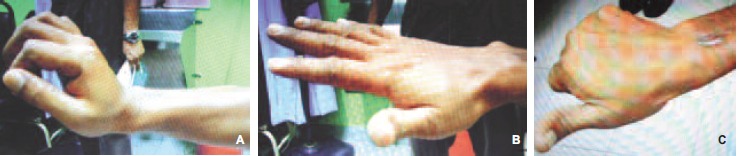# Is A New Combination of Tendon Transfers For Radial Nerve Palsy (RNP) Needed?

**DOI:** 10.5704/MOJ.1403.006

**Published:** 2014-03

**Authors:** IMA Ramdhan, SA Nawfar, M Paiman

**Affiliations:** Department of Orthopaedics, Sultanah Nur Zahirah Hospital, Kuala Terengganu, Malaysia; Department of Orthopaedics, Universiti Sains Malaysia Hospital, Kota Bahru, Malaysia; Department of Orthopaedics, Universiti Sains Malaysia Hospital, Kota Bahru, Malaysia

## Abstract

Radial nerve palsy following traumatic humeral diaphyseal fractures occurs uncommonly. Most recover with good outcome. However the cases with poor outcome or recovery will have tendon transfers to regain the lost functions. Various tendon transfer methods and choices are available in literatures citing acceptable to good results. We report a case of radial nerve transection secondary to a closed traumatic diaphyseal fracture of the humerus which did not improve despite the repair. As many conventional technique produce asynergistic muscle action with secondary post-transfer deformity, thus we had resorted to tendon transfer procedure using an unconventional donor and recipient tendons yielding good results. This new combination of tendon transfers was invented involves better dynamic correlation of synergistic muscles action and produces good outcome and hand function.

**Key Words:**

Tendons transfer, radial nerve palsy

## Introduction

Radial nerve palsy is an uncommon presentation with humeral diaphyseal fractures. The radial nerve lies in contact with periosteum in the mid-diaphysis of the humerus; therefore it is commonly injured in fracture of the middle third of the shaft of the humerus1. About 10% to 17% humeral fractures will present with radial nerve injury. When it occurs in closed diaphyseal fractures it is most often a neurapraxia and early recovery is anticipated. These cases can be managed non-operatively. Unfortunately certain fracture with delayed union do end up with or without recovery of the radial nerve. The delay in nerve recovery could be due to nerve being transected or crushed during the time of injury. Therefore nerve exploration is performed in some cases when there is no sign of spontaneous recovery within 3 months. If the nerve is transected and repairable, end to end repair will be attempted. If the nerve is not repairable, other nerve procedures, like nerve transfer or nerve grafting, will be considered.

Patients with high energy injury to the arm are at high-risk to have severe injury to the radial nerve, either severe contusion, laceration or Neurotmesis. They are highly likely to have unfavorable nerve recovery despite surgical intervention with reconstruction of the radial nerve. Thus, early tendon transfers have been proposed in this group of patients in order to maintain wrist, thumb and fingers extension.

Tendon transfer usually is the last option in managing a patient with radial nerve palsy if there is no nerve recovery after nerve surgery. The tendon transfer must follow its prerequisite criteria and principles, and it requires multidisciplinary approach, which includes the orthopaedic surgeon, hand rehabilitation therapist, as well as full participation by the patient. However, the existing combination of tendon transfers use asynergistic muscles action (as in Tsuge procedure or Boyes transfer) as flexor tendon of the wrist is re-educated to become finger extensors while patient forms a grasping or a fist (finger extends when flexion is needed). These conventional techniques produce asynergistic muscle action with secondary post-transfer deformity or weakness. Therefore, the case below illustrates the problems of a closed diaphyseal fracture which had radial nerve transection and which was later repaired but with poor recovery and subsequently managed with a nonconventional tendon transfer using combination of synergistic muscle action, resulting in good outcome and hand function.

## CASE REPORT

**Fig. 1: A. Plain radiograph of right humerus in May 2009. B. Plain radiograph of right humerus in Feb 2010.**
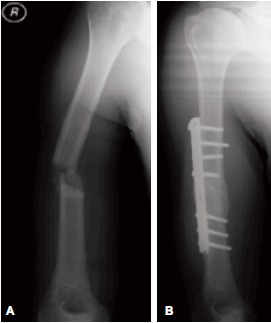


A 29-year old right handed gentleman working as a forklift operator was referred to Hospital USM for further management of his right upper limb problem. He had been involved in an industrial accident in March 2009 in which he had sustained closed fracture of the mid-shaft of the right humerus associated with radial nerve palsy. We reviewed him in May 2009 and noted there was no sign of radial nerve recovery (no wrist, thumb and fingers extension) with sign of non-union of the right humeral fracture on the plain radiograph (Figure 1A). Therefore, surgery was performed with plating of the right humeral fracture and the fracture site augmented with bone graft. The radial nerve was explored and found to be transected, and neurorrhaphy (perineural repair technique) was performed.

At 10 months post-operation, the right humeral fracture was well united (Fig.1B) but without recovery of radial nerve injury (no advancement of Tinel sign, with no return of wrist, thumb and fingers extension). Tendon transfer was performed in June 2010 with three donor and three recipient muscles involved [Pronator Teres (PT) to Extensor Digitorum Communis (EDC); Flexor Carpi Radialis (FCR) to Extensor Pollicis Longus (EPL); Flexor Digitorum Superficialis (FDS) of ring finger to Extensor Carpi Radialis Brevis (ERCB)]. The Pulvertaft weave method was used to attach the tendons. The hand then was immobilized with the wrist in about 45 degrees extension, fingers and thumb in full extension and the metacarpo-phalangeal (MCP) joints in 90 degrees flexed position, for a month. Hand rehabilitation programmes was started by the hand therapist including post-tendon transfer muscle re education. This patient had attended therapy only once every two weeks.

In October 2011 (about 16-month post-tendon transfer), the patient was able to perform active wrist extension > 29°, full extension of metacarpophalangeal joint and first web space opening span was 35^z^ (Figure 2). He was fully satisfied with the outcome of the surgery and had returned to work. The functional Bincaz score was 8^1^; scoring the outcome as excellent.

## Discussion

Most of the cases of the radial nerve injury after humeral shaft fracture are neurapraxia^2^. Neurapraxia is local segmental demyelination causes temporary blockade of nerve impulse transmission. It takes about 8 to 12 weeks to recover with a process of remyelination^2^. Therefore, the sign of spontaneous recovery is expected in neurapraxia within the first 3 months post-injury. If there is no sign of spontaneous recovery, the nerve exploration is indicated in order to find out the nerve status whether it is still in continuity or transected. If the nerve is still in continuity, it means the possibility of axonometsis with chances of recovery. If the nerve is transected, it means neurometsis and nerve repair must be done without tension. Axonometsis is when there is an injury to the axon within intact endoneural tube. The distal portion of injured axon will undergo Wallerian degeneration that begins within hours post-injury and completes by 6-8 weeks^2^. The proximal stump of the axon will proliferate and regenerate the new axon distally within the endoneural tube until reach the target tissues. The estimation rate of the nerve regeneration is about 1mm/day or 1in./month^2^. Neurometsis is when there is an injury to the axon as well as to the endoneural, perineural and epineural tubes, or when there is total cut of the nerve. The distal portion of the injured axon and nerve will undergo Wallerian degeneration (similar to the axonometsis) and the proximal stump again will regenerate new axon. However, as there is no intact neural tube, the nerve regeneration from the proximal stump may loss its direction and divert to the surrounding soft tissues if left untreated^2^. The nerve transfer and nerve grafting should be considered if the nerve is not repairable or presence of large nerve gap^2^. In general, the non-healing nerve injury is treated by the ‘step-ladder’ approach in which the nerve procedures before any soft tissue procedures. The nerve procedures include external neurolysis, nerve end-to-end repair, nerve grafting and nerve transfer^2^. Any nerve procedures should be performed within 6-month post-injury in order to give time for reinnervation process before the motor-end-plate degenerates at about 12 to 18 months post injury^2^. The prognostic factors of nerve recovery are location or site of the injury, type of nerve injury, age of the patient, severity or energy of the injury, injury to surgery interval as well as technique of the nerve repair^2^. In the case above, the radial nerve was transected and the end-to-end nerve repair was performed 2-month post injury. Perhaps a nerve conduction study if done earlier would have revealed the calamity to the nerve earlier.

Later on at follow up due to no presence of recovery despite giving adequate time soft tissue procedure or tendon transfer was considered for the case. There are principles of tendon transfer have to be followed ^3^. The recipient site should be free from scar tissue with adequate soft tissue cover to provide gliding surface for the tendon transfer. The joints affected by the tendon transfer must be supple and free from contractures. Furthermore, the donor muscles should have adequate muscle power or strength because the muscle power will reduce after transfer. The muscle power must be at least MRC 4 before transfer. It must also be expendable (will not create major functional impairment after transfer), have sufficient amplitude and excursion with voluntary control. Preferably the donor muscle has synergistic function, for example flexor of the wrist works simultaneously with extensor of the fingers, and extensor of the wrist works simultaneously with the flexor of the fingers. During the transfer should ensure the line of pull as straight as possible to optimize the working of the transfer. This is because any turn or pully-assisted movement may weaken the tendon transfer. The correct tension applied is also important during surgery to provide better function. The main aim of tendon transfer in radial nerve palsy is to achieve wrist as well as finger and thumb extension^3,4,5^ .

There various methods to perform tendon transfer in radial nerve palsy. One of the preferred methods is a modified Tsuge procedure, using the PT to restore wrist extension, the FCR for fingers extension and PL for thumb extension in association with a tenodesis of the abductor pollicis longus (APL) to the brachioradialis to achieve thumb abduction as well^4^. Other famous transfer is Boyes transfer that transferring FDS of middle and ring finger to the EDC and EPL (to restore finger and thumb extension), as well as PT to ECRB as mentioned before^3,4^.

In the illustrated case, the surgeon had used a modified tendon transfer technique with transferring PT to EDC to restore fingers extension, FCR to EPL for thumb extension, and FDS of ring finger to ECRB for wrist extension. This method is a modification of the fore mentioned method producing an equally good result. The commonly used donor tendons were utilized to attain different functions. The FDS to the ring finger was particularly used for wrist extension in order for it to function in synchronized manner i.e. the wrist extends when the patient forms the grip. FCR was used in this transfer in order to avoid deactivation of FCU that may lead to radial deviation of the wrist as well as reduces the grip strength^4^. Furthermore, in some maneuvers, for instance hammering that required strong wrist flexor and ulnar deviator, may be affected if FCU is chosen for the transfer 5. Other advantage of using the FCR for the thumb extension is because the FCR is also radial deviator hence the action is nearly similar to a thumb extension and abduction (synergy in action hence easier to train patients). ECRB is the preferred tendon of attachment because it is the strongest wrist extensor with the lowest radial deviation moment arm. The PT was used as a finger extensor as the action to grasp or grab objects starts with a pronated forearm (again synergy with the physiologic requirement).

Despite all measures the most important management is the post-transfer therapy in order to ensure the successful outcome tendon transfers. It consists of post-operative rehabilitation exercises, scar management as well as muscletendon re-education^5^. The hand was in this case immobilized in the position of the wrist in 45 degrees of extension, the MCP in 90 degrees of flexion and the interphalangeal (IP) joints are in extension. The thumb is extended and abducted within the splint. The immobilization was continued for four to five weeks before the formal occupational therapy session started. The splint was then worn between the occupational therapy sessions for additional four to six weeks. The main aim of scar management was for soft tissue mobilization in order to minimize the formation of deep scar that may lead to peritendinous adhesion at the repair site. The muscletendon re-education was to educate the patient on how to use their transfers with ease.

The surgeon, hand therapist as well as full participation by the patient play the important role to determine the successful of tendon transfer procedure. In the illustrated case, this patient achieved good wrist, fingers and thumb extension after about 16-month post-transfer even only attending the therapy session once every two weeks. This new combination of tendon transfers did not require close therapist supervision, thus suitable for patients with poor access for physiotherapy, poor frequency of therapy and poor follow-up with inexperienced therapist pertaining to tendon transfer rehabilitation.

Bincaz had described the assessment of functional results of post tendon transfer in radial nerve palsy (Table I) 1. A scoring system with a score of ≥ 8 points was considered Excellent, 6 or 7 points was considered Good, 4 or 5 points as Fair, and points ≤ 3 as Bad.

In the illustrated case, the patient was able to perform wrist extension > 29°, full extension of metacarpophalangeal joint and first web space opening between 30 to 39°. He is fully satisfied with the outcome of the surgery and has already returned to work. These made the score is 8 points, showing the functional outcome is Excellent.

This modified tendon transfer technique can be used as one of the methods for the reconstruction of wrist, fingers and thumb extension in patients with radial nerve palsy with favourable outcome.

**Table I: Bincaz score for assessment of tendon transfers for radial nerve palsy:**
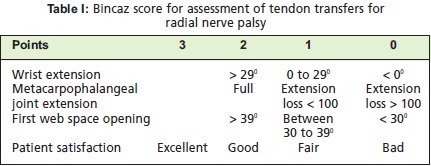


**Fig. 2:Patient was able to do wrist extension (A), fingers extension (B) and thumb extension (C).**